# Predictive Value of Several Parameters for Severity of Acute Pancreatitis in a Cohort of 172 Patients

**DOI:** 10.3390/diagnostics15040435

**Published:** 2025-02-11

**Authors:** Florina Alexandra Liță (Cofaru), Irina Anca Eremia, Silvia Nica, Lăcrămioara Aurelia Brîndușe, Narcis-Octavian Zărnescu, Alexandru Constantin Moldoveanu, Loredana Gabriela Goran, Carmen Fierbințeanu-Braticevici

**Affiliations:** 1 Internal Medicine II and Gastroenterology Department, Emergency University Hospital Bucharest, Carol Davila University of Medicine and Pharmacy, 050474 Bucharest, Romania; florina.cofaru@drd.umfcd.ro (F.A.L.); alexandru.moldoveanu@umfcd.ro (A.C.M.); loredana.goran@umfcd.ro (L.G.G.); carmen.fierbinteanu@umfcd.ro (C.F.-B.); 2 Emergency Department, Emergency University Hospital Bucharest, 050098 Bucharest, Romania; silvia.nica@umfcd.ro; 3 Department of Family Medicine III, Carol Davila University of Medicine and Pharmacy, 050474 Bucharest, Romania; 4 Department of Emergency and First Aid, Carol Davila University of Medicine and Pharmacy, 050474 Bucharest, Romania; 5 Department of Public Health and Management, Carol Davila University of Medicine and Pharmacy, 020021 Bucharest, Romania; lbrinduse@gmail.com; 6 Department of General Surgery, Carol Davila University of Medicine and Pharmacy, 050474 Bucharest, Romania; narcis.zarnescu@umfcd.ro; 7 Second Department of Surgery, Emergency University Hospital Bucharest, 050098 Bucharest, Romania

**Keywords:** acute pancreatitis, severity score, predictability, CRP, VAS scale

## Abstract

**Background**: The optimal management of patients with acute pancreatitis is directly related to the early detection of the mild, moderate, or severe forms of the disease, which remains a continuous challenge despite the availability of various severity scores. The aim of this study was to identify prognostic factors with the highest predictive value specific to the local patients and elaborate the score to identify the severe cases. **Materials and Methods**: A retrospective observational cohort study included 172 patients diagnosed with acute pancreatitis. Personal, clinical, laboratory, and imaging factors and their influence on the severity of acute pancreatitis were evaluated. **Results**: Etiology nonA-nonB (any etiology except unique alcoholic or biliary etiology), presence of diabetes mellitus, the pain Visual Analogue Scale (VAS), White Blood Cells (WBCs), and CRP (C-reactive protein) levels were found to be directly associated with the severity of acute pancreatitis (AP). Prediction scores were calculated to estimate disease severity using the following regression equations: Prediction Acute Pancreatitis Severity (PAPS) score I = 1.237 + 0.144 × nonA-nonB (0 = no, 1 = yes) + 0.001 × WBC1 + 0.027 × VAS0 and PAPS score II = 1.189 + 0.001 × CRP (mg/L) + 0.135 × nonA-nonB etiology (0 = no, 1 = yes) + 0.025 × VAS0 − 0.047 × CA1. The PAPS Score II demonstrated the best performance. At a cut-off value of 1.248, the score had 80% sensitivity, 80.9% specificity, a positive predictive value (PPV) of 28.6%, a negative predictive value (NPV) of 97.7%, and an accuracy of 80.8%. For a cut-off value of 221.5 mg/L, the accuracy of CRP was 81.4% for predicting severe AP. **Conclusions**: The PAPS score II is an easy-to-use, fast, and affordable score for determining cases of severe disease for patients diagnosed with AP.

## 1. Introduction

Acute pancreatitis (AP) is one of the most usual gastrointestinal pathologies that lead to hospital admission. The incidence rate is variable from one region to another. There are countries with a rate of 15/100,000, like Denmark, or other countries that register a much higher rate of incidence, such as Sweden, with 83.7/100,000 [1]. Globally, the incidence rate, the mortality rate, and the disability-adjusted life years (DALY) due to acute pancreatitis are increasing [1]. The highest age-standardized incidence rate was reported for Eastern Europe in a recent study [2]. Approximately 20% of the cases of acute pancreatitis develop a severe form of disease with a mortality rate ranging between 20 and 40% [3]. Severe forms of disease necessitate intensive care unit (ICU) measures, high costs, and a lethal potential in spite of all the resources involved. Therefore, the prognosis of severity for acute pancreatitis is a common subject of study.

Until now, several classifications of severity have been implemented. The Revised Atlanta Classification from 2012 is the most frequently referenced. It involves local and systemic factors like necrosis and organ failure. There are three grades of severity: mild, moderate, and severe, based on different combinations of these factors [4].

The Revised Atlanta Classification improves upon the original Atlanta Classification from 1992 by introducing clearer clinical and radiologic terminology [5]. AP is classified as either interstitial edematous or necrotizing with four different pancreatic or peripancreatic fluid collections described: acute peripancreatic fluid, pancreatic pseudocyst, acute necrotic collections, and walled-off necrosis. Severity is graded as mild, moderate, or severe based on organ failure and complications; the mild form of acute pancreatitis has no organ failure involved with no complications, the moderately severe form is about a transient organ failure (48 h), and/or local or systemic complications, and the severe form presents a persistent simple or multiple organ failure (>48 h) [6].

The Determinant-Based Classification of Acute Pancreatitis Severity was developed through a global web-based survey and an international symposium with the contribution of professionals from different disciplines. This classification is based on local determinants (pancreatic or peripancreatic necrosis present or not, infected or not) and systemic determinants (presence or absence of organ failure transient or persistent). It categorizes severity into four grades of severity: mild, moderate, severe, and critical.

This disease has diverse clinical manifestations with variable outcomes. Due to pancreatitis’ heterogeneity, finding a severity score that differentiates the potentially severe cases from the potentially mild ones as early as possible to ensure optimal management has always been a desiderate. The most widely used severity scores are the Ranson score and APACHE II (Acute Physiology and Chronic Health Enquiry II) [5]. The Ranson score was the first severity score system developed for acute pancreatitis, introduced in 1974. It uses 11 parameters and can be determined in 48 h. However, the Ranson score has limited predictive power for severe cases of pancreatitis [3].

Part of the Ranson criteria is collected when the patient is admitted to the hospital (age, glucose, LDH, AST, and WBC), and the others are collected after 48 h (serum calcium, base deficit, hematocrit, BUN, sequestrated fluid, and hypoxemia). A score of ≥ 3 indicates a severe form of AP [7].

APACHE score was initially used to identify patients requiring ICU admission. This score involves 12 parameters and is determined within 24 h [3].

For SIRS, four parameters were followed: temperature (>38 °C or <36 °C), heart rate (>90 bpm), respiratory rate >24 breaths/minute or PCO2 < 32 mmHg, and WBC (<4000 or >12,000/mm^3^). When all parameters are encountered, a severe form of disease is revealed. Criteria used for BISAP were collected during the first 24 h of admitting the patients. These were urea, Glasgow Coma Score (GCS), evidence of SIRS, age, and presence of pleural effusion. Some validation studies proved similar results in predicting mortality with the BISAP score in comparison to the APACHE II score [7,8,9]. Yet BISAP is not validated for predicting neither the need for intensive care or intervention nor the length of hospital stay [7,8,9]. HAPS uses a different perspective; it identifies patients with acute pancreatitis who do not need intensive care. This score is built up by three variables: normal serum creatinine level, normal hematocrit level, and the lack of guarding and/or rebound tenderness. Therefore, patients with mild forms of acute pancreatitis can be spotted in the very first 30 min. This score is easy to use, cheap, and quick [7]. At first, describing the Multiple Organ Dysfunction Syndrome was counting how many systems were failing. Recently, descriptive numeric scales that quantify organ dysfunction were developed. The Multiple Organ Dysfunction Score uses a scale with physiologic criteria. Six organ systems are considered: respiratory, renal, hepatic, cardiovascular, hematologic, and neurologic. For the respiratory system, the PO2/FIO2 ratio is calculated, no matter the mode of mechanical ventilation used or the level of Positive End-Expiratory Pressure (PEEP). For the renal system, serum creatinine level is measured in μmol/liter, indifferent to the use of dialysis. The hepatic system is represented by the serum bilirubin level measured in μmol/liter. The R/P ratio is the product of the heart rate and right atrial pressure divided by the mean arterial pressure and stands for the cardiovascular system. Platelet count reveals the hematologic system function. GCS indicates the neurologic function [10]. Each parameter is noted from zero to five points, depending on the grade of dysfunction. The risk of mortality increases proportionally with the overall degree of dysfunction [10].

Imaging investigations are part of the assessment of a patient with acute pancreatitis. Abdominal ultrasound is a routine investigation for all patients with acute pancreatitis due to its high sensitivity (90–95%) on the diagnosis of biliary lithiasis [11]. The abdominal ultrasound is also useful in assessing the pancreatic parenchyma, peripancreatic tissues, the biliary tree, and regional vascular structures [12]. In some cases, complications such as peripancreatic fluid collections, arterial pseudoaneurysm, or venous thrombosis can be identified through sonography [12]. Sometimes, the pancreas may appear normal on an ultrasound. Chest X-rays are performed to rule out a perforated duodenal ulcer and to identify pleural effusion or infiltration, which are commonly associated with poor outcomes [11,13,14] or respiratory dysfunction. These X-ray findings contribute to assessing the severity of AP. Abdominal X-rays are performed in some of the patients for differential diagnosis or to detect ileus, which indicates intestinal organ failure and may reflect disease severity [13]. Abdominal CT scan provides valuable information on pancreatic inflammation, pancreatic necrosis, and the grade of organ dysfunction. These findings correlate with the likelihood of complications and the outcome and informed treatment decisions (antibiotic therapy, interventions) [13].

As pain is one of the primary reasons for hospital presentation in patients with AP, pain assessment is crucial not only for guiding pain management therapy but also for assessing the progression of the disease and the outcome. The VAS (Visual Analogue Scale) was first time used in 1923 by Freyd in psychology and originated in the Scott Co’s laboratory in 1920 [15].

As in the case of any other pathology, associated comorbidities play an important role in the evolution of the disease. The Charlson Comorbidity Index (CCI) predicts a 10-year survival in patients with multiple comorbidities. The CCI considers factors such as age, history of myocardial infarction or the probability of having such a history (determined by electrocardiogram (ECG) and/or enzyme changes), congestive heart failure, peripheral vascular disease, the history of transient ischemic attacks or cerebrovascular accident, dementia, chronic obstructive pulmonary disease (COPD), peptic ulcer disease, connective tissue disease, liver disease, diabetes, hemiplegia, moderate or severe chronic kidney disease, the presence of a solid tumor, leukemia, lymphoma, and autoimmune disease syndrome (AIDS) [16]. Using this index can bring valuable information on the long-term prognosis.

Multiple cohort studies are being conducted to identify the optimal severity score (easy to use, few resources needed, financially advantageous, rapid resolution, and with good predictive power) in a specific region or institution. Factors such as geographical region, type of hospital, local protocols and procedures, local clinical practice, and the specific patient population addressed to a medical facility contribute valuable insights, even in small, local studies

The present study aimed to assess the prognostic value of several clinical and paraclinical parameters in establishing the severity of AP and to elaborate a new score in predicting the evolution of this pathology.

## 2. Materials and Methods

A retrospective study was implemented at the Emergency University Hospital of Bucharest in Romania after receiving approval from the local Ethics Committee. The inclusion criteria were as follows: adult patients (>18 years old) admitted to the hospital (to surgical, gastroenterological, or internal medicine wards) with the diagnostic of acute pancreatitis (AP). According to the American College of Gastroenterology: ‘The diagnosis of AP most often is established by identification of 2 out of 3 following criteria: (i) abdominal pain consistent with the disease, (ii) serum amylase and/or lipase greater than 3 times the upper limit of normal, and/or (iii) characteristic findings from abdominal imaging’ [6,17]. All patients underwent clinical assessment, laboratory tests, and imaging investigations. Comprehensive data were recorded for each patient, including demographic data like sex and age, hospitalization days, ward of admission, days spent in the intensive care Unit (ICU), etiology, obesity, smoking addiction, Body Mass Index (BMI), comorbidities, particular cases of pregnancy or confinement, other medical history, pain scale, blood pressure, ventricular allura on admission, data related to organ failure or Multiple Organ Dysfunction Syndrome (MODS), treatment, temperature records, cultures, surgical records, complications, Endoscopic retrograde cholangiopancreatography (ERCP) procedure, and outcome. All the patients provided written informed consent, and the study complied with the Ethical Principles for Medical Research Involving Human Subjects in the World Medical Association Declaration of Helsinki.

Laboratory tests included white cell count, hemoglobin, hematocrit, blood glucose, urea, creatinine, liver function tests, bilirubin levels, serum amylase, serum lipase, calcium, triglycerides, cholesterol, C-reactive protein, pH, the international normalized ratio (INR), and cultures. Except for cultures, all blood tests were performed upon hospital admission. Follow-up blood tests were conducted at 24 h post admission for hemoglobin, hematocrit, urea, creatinine, liver function tests, bilirubin levels, serum amylase, and serum lipase. A third set of tests was performed 48 h post-admission concerning calcium and repeated liver function tests, bilirubin levels, serum amylase, and serum lipase.

Imaging investigations were also performed on all patients. All the patients had at least two imaging tests: an abdominal ultrasound and a chest X-ray. Abdominal X-rays were not considered in this study due to the low number of patients undergoing this test. Clinical exams and chest X-rays were typically sufficient, rendering abdominal X-rays unnecessary in most cases. Approximately 60% of patients had their abdomen Computed Tomography (CT) scanned. Of these, 20% underwent a second CT scan to assess disease progression, identify complications and guide treatment decisions.

From an etiological perspective, patients were classified into 7 categories: patients with acute pancreatitis of biliary etiology, alcoholic etiology, hypertriglyceridemia, mixed etiology (biliary and alcoholic), mixed etiology (alcoholic and hypertriglyceridemia), other etiologies, and idiopathic acute pancreatitis.

BMI was calculated as body weight (measured in kilograms) divided by the square of body height (m^2^).

The Visual Analogue Scale (VAS) was used to evaluate the pain of the studied patients. VAS consists of a straight line drawn from the “no pain at all” endpoint to the “pain as bad as it could be” endpoint. Patients mark down on the line the level of pain experienced at that particular moment. Measuring the distance between “no pain at all” and the mark left by the patient on that specific line defines the level of pain experienced by the patient [18]. In our study, a 1 to 10 numerical scale was added to VAS in order to evaluate pain. Pain levels were assessed at four intervals: upon hospital admission and 24, 48, and 72 h post-admission.

While registering the comorbidities of the studied patients, the Charlson Comorbidity Index (CCI) was calculated for each patient.

In this study, the 2012 Revised Atlanta Classification was used as the gold standard for stratifying patients according to the severity of acute pancreatitis. Although we initially considered both the Revised Atlanta Classification and the Determinant-Based Classification, we chose the Revised Atlanta Classification due to the fact that it is the most widely used classification method worldwide, being broadly accepted in clinical practice and frequently encountered in the specialized literature.

Several severity scores were used to identify patients at higher risk of progressing to a severe form of AP, like Ranson Score, Systemic Inflammatory Response Syndrome (SIRS), Bedside Index for Severity in Acute Pancreatitis (BISAP), Harmless Acute Pancreatitis Score (HAPS), Multiple Organ Dysfunction Score (MODS). We calculated only those scores for which the necessary data were available for all enrolled patients. The APACHE II score could not be calculated for all the patients since some of the data needed were not available outside the ICU.

Of all the scores calculated for our patient cohort, we selected Ranson and BISAP for direct comparison with the new models (CRP, PAPS I, and PAPS II), as these are some of the most established systems for assessing the severity of acute pancreatitis and are widely used in clinical practice and the literature. This comparison allows for a more robust validation of our new model against the most widely recognized existing scores.

After the analysis of distribution for continuous variables using the Kolmogorov–Smirnov test, the mean and standard deviation were calculated for all normally distributed variables and the median and range interval for non-normal distributed variables. The comparison between mild and severe AP was analyzed using parametric (*t*-test) and non-parametric (Mann–Whitney test) tests according to the distribution of variables.

The qualitative variables were presented as counts and percentages, and the chi-square or Fisher’s exact test was used for the comparison between mild/moderate and severe AP.

Spearman’s rho correlation coefficient was used to assess the correlation between the severity of AP and different factors that can influence the severity of AP (gender, age, etiology, serological tests, etc.).

To assess the independent predictors of AP severity, the logistic regression analysis was used. Linear multiple regression was used to develop models to predict the severity of AP and to calculate the scores.

The receiver operating characteristics (ROCs) were used to evaluate the diagnostic performance of the developed models. The sum of sensitivity and specificity for each value was calculated, and the higher sum was used to establish the cut-off value for each of the models. Also, the validity (sensitivity, specificity), predictive values (positive and negative), and accuracy were calculated for each model: CRP, PAPS 1, PAPS 2 (new models), Ranson, and BISAP, including 95% confidence interval (95% CI). For all the models, the area under the curve (AUC) and 95% CI were calculated, and according to AUC values, the performance of the models was compared.

A *p*-value < 0.05 was considered statistically significant.

Data analysis was performed using Statistical Package for Social Sciences (SPSS) 29.0 version (IBM SPSS Statistics for Windows, Version 29.0. Armonk, NY, USA: IBM Corp).

## 3. Results

### 3.1. Patient Cohort

This retrospective study involved 172 patients diagnosed with AP who were admitted to the hospital from the emergency room (ER) to a gastroenterological (31.39%), internal medicine (26.74%), or surgical department (41.86%) over the course of 4 years.

A majority of patients (55.23%) presented to the hospital early, within the first 24 h from symptom onset. A further 28.48% sought medical attention between 24 and 72 h after symptom onset, while 16.27% delayed their presentation to the hospital for at least 72 h.

The length of hospitalization ranged between 2 and 69 days, with an average stay of 11.87 days.

A total of 91.3% (*n* = 157) of the patients included in this study had mild and moderate forms, while 8.7% (*n* = 15) presented with severe forms. The analysis of the general characteristics of the patients (Table 1) revealed no statistically significant differences in gender distribution (*p* = 0.588), with males predominating in both groups: 60.5% (*n* = 95) in mild and moderate AP and 53.3% (*n* = 8) in severe AP.

Similarly, no significant variations were observed with respect to age (mean ± standard deviation: 55.9 ± 16.2 years in mild and moderate AP and 59.7 ± 18.0 years in severe AP, *p* = 0.388).

Regarding age distribution, 43.02% of patients were over 60 years old, 39.53% were between 40 and 60 years, and 17.44% were aged between 18 and 40 years. The youngest patient is 18 years old, and the oldest is 93 years old.

### 3.2. Etiological Distribution

Biliary lithiasis is the most common cause of AP in the studied group, followed by alcohol consumption and hypertriglyceridemia (Table 2). A total of 87% of the studied patients had a unique etiology, while the rest encountered multiple etiologies (Table 3). The biliary etiology was met both as a unique etiology and in combination with alcohol consumption (Table 4).

An important finding highlighted by the analysis was the association between the etiology of pancreatitis and disease severity (Table 1). Biliary etiology was more frequently observed in mild and moderate AP cases (43.3% vs. 26.7%, *p* = 0.012), whereas nonA-nonB etiology was more commonly associated with severe forms (53.3% in severe AP vs. 19.7% in mild and moderate AP, *p* = 0.007). These findings were supported by correlation analysis (Table 5), which demonstrated a significant positive correlation between nonA-nonB etiology and disease severity (r = 0.226, *p* = 0.003). Although alcoholic etiology was more prevalent in mild and moderate AP (36.9%) compared to severe AP (20.0%), this difference did not achieve statistical significance (*p* = 0.152).

### 3.3. Metabolic Factors and Comorbidities

Body mass index (BMI) did not differ significantly between groups (25.0 kg/m^2^ in mild and moderate AP vs. 26.0 kg/m^2^ in severe AP, *p* = 0.497), as shown in Table 1. However, the presence of diabetes mellitus (DM) was identified as a significant factor associated with disease severity (Table 1), being more prevalent among patients with severe AP (46.7% vs. 19.7%, *p* = 0.016). The correlation between diabetes mellitus and severity was further supported by the Spearman coefficient in Table 5, which demonstrated a significant positive association (r = 0.183, *p* = 0.016). In contrast, a history of acute pancreatitis, reported in 28.7% of patients with mild and moderate AP and 26.7% of those with severe AP, showed no statistically significant differences (*p* = 0.870), indicating that prior episodes do not directly influence disease severity.

### 3.4. Clinical and Biological Parameters

In terms of symptom severity, patients with severe AP reported significantly higher pain levels on the VAS scale, with a median score of 10.0 (9.0–10.0) compared to 8.0 (7.0–10.0) in the mild and moderate AP group (*p* = 0.003). The correlation between VAS scores and disease severity was confirmed by statistical analysis (Table 5), showing a significant positive correlation (r = 0.229, *p* = 0.003).

From a biological perspective, white blood cell (WBC) count and C-reactive protein (CRP) levels were significantly higher in patients with severe AP (Table 1 and Table 5). Median WBC levels were 19,080/mm^3^ in severe AP compared to 12,280/mm^3^ in mild and moderate AP (*p* = 0.037), while CRP levels had medians of 223.0 mg/L (severe AP) vs. 85.0 mg/L (mild and moderate AP, *p* = 0.002). Correlation analysis confirmed these associations (Table 5), with CRP demonstrating a moderate positive correlation with disease severity (r = 0.233, *p* = 0.002) and WBC showing a weaker but significant correlation (r = 0.159, *p* = 0.037). In contrast, calcium markers exhibited a significant negative correlation with severity (r = −0.188, *p* = 0.014).

Other biological parameters, including ALT, amylase, lipase, and fluids administered during the first 24 h, did not show significant correlations with disease severity (*p* > 0.05, Table 1 and Table 5).

### 3.5. Independent Predictors of Pancreatitis Severity

Regression analysis (Table 6) identified independent predictors of AP severity. NonA-nonB etiology was associated with a significantly increased risk of severe disease (B = 1.536, *p* = 0.006, OR = 4.645, 95% CI: 1.565–13.786). Diabetes mellitus also emerged as an independent predictor (B = 1.269, *p* = 0.022), tripling the risk of severe forms (OR = 3.556, 95% CI: 1.198–10.555). Pain intensity, as measured by the VAS score, was linked to a doubling of severity risk (B = 0.742, *p* = 0.011, OR = 2.099, 95% CI: 1.184–3.721).

C-reactive protein was an important biological marker associated with a marginal yet significant increase in risk (B = 0.009, *p* = 0.001, OR = 1.009, 95% CI: 1.003–1.014). Lower levels of calcium had a protective effect (B = −0.642, *p* = 0.018, OR = 0.526, 95% CI: 0.309–0.896). Parameters such as sex, age, history of pancreatitis, amylase, and lipase levels did not show significant associations with disease severity (*p* > 0.05).

Using multivariable linear regression, variables associated with AP severity were identified. The parameter demonstrating the strongest association with AP severity was CRP. Based on the results, three predictive models were developed. The first model includes only CRP as a predictor of AP severity. The second model excludes CRP and incorporates parameters with statistical significance (nonA-nonB etiology, baseline VAS score [VAS0], calcium, and initial WBC count [WBC]). The third model combines CRP with parameters that retained statistical significance alongside CRP (nonA-nonB etiology and VAS0) (Table 7).

Using the variables significantly associated with AP severity, prediction scores can be calculated to estimate disease severity.

The regression equation for the CRP-only model is AP Severity = 0.993 + 0.001 × CRP (mg/L).

The Prediction Acute Pancreatitis Severity (PAPS) Score I, which excludes CRP, is calculated using the following regression equation: PAPS Score I = 1.237 + 0.144 × nonA-nonB etiology (0 = no, 1 = yes) + 0.001 × WBC1 + 0.027 × VAS0.

For the third model, the PAPS score II, the regression equation is PAPS score II = 1.189 + 0.001 × CRP (mg/L) + 0.135 × nonA-nonB etiology (0 = no, 1 = yes) + 0.025 × VAS0 − 0.047 × CA.

The CRP cut-off value was established at 221.5 mg/L, with a sensitivity of 53.3%, specificity of 84.1%, positive predictive value (PPV) of 24.2%, negative predictive value (NPV) of 95%, and an accuracy of 81.4%.

At a cut-off value of 20.5, the performance of PAPS score I is detailed in Table 8.

Among the three models, the PAPS score II demonstrated the best performance. At a cut-off value of 1.248, the PAPS score II achieved a sensitivity of 80%, specificity of 80.9%, PPV of 28.6%, NPV of 97.7%, and an accuracy of 80.8% (Table 8).

The BISAP score and Ranson score were calculated for the studied cohort, and the following results were obtained. For the BISAP score, it had 60% sensitivity, 83.4% specificity, 25.7% PPV, 95.6 NPV for predicting severity, and an accuracy of 80.8%. The Ranson score proved to predict severity for cases of acute pancreatitis with a sensitivity of 53.3%, a specificity of 68.2%, a PPV of 13.8, and an NPV of 93.9. The accuracy of the Ranson score was 66.9%.

**Figure 1:**
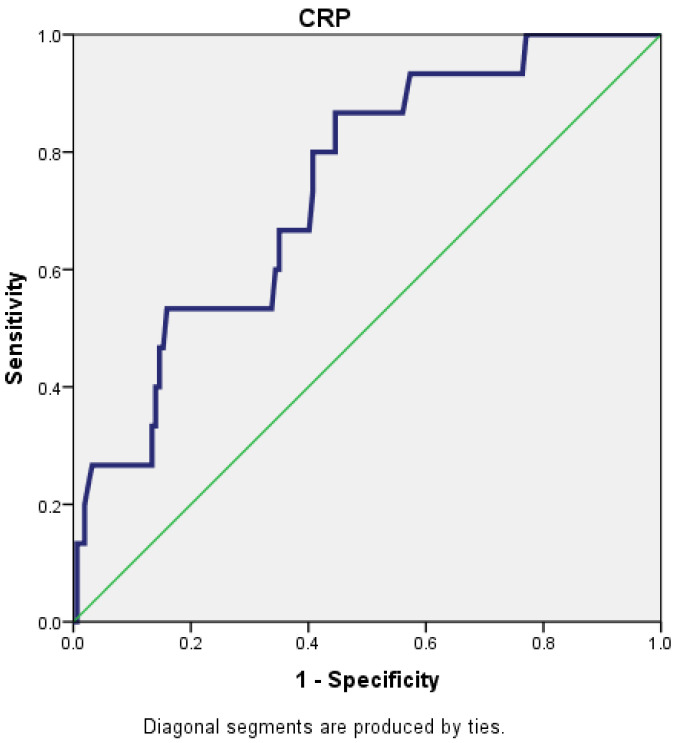
Receiver operating characteristic (ROC) curve for CRP.

For a cut-off value of 221.5 for CRP, the AUC (area under the curve) was 0.739 (95% CI: 0.619–0.859), which reflects that CRP is a fair predictor for the severity of AP (Figure 1).

**Figure 2:**
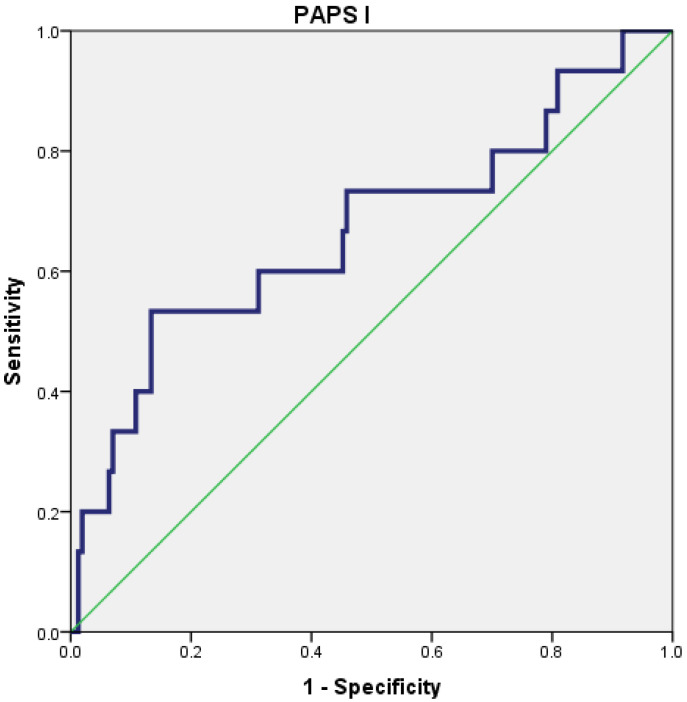
Receiver operating characteristic (ROC) curve for PAPS I.

The PAPS score I had a poor predictive value for the severity of AP (AUC = 0.667; 95% CI = 0.502–0.832), with the best cut-off value being 20.5 (Figure 2).

**Figure 3:**
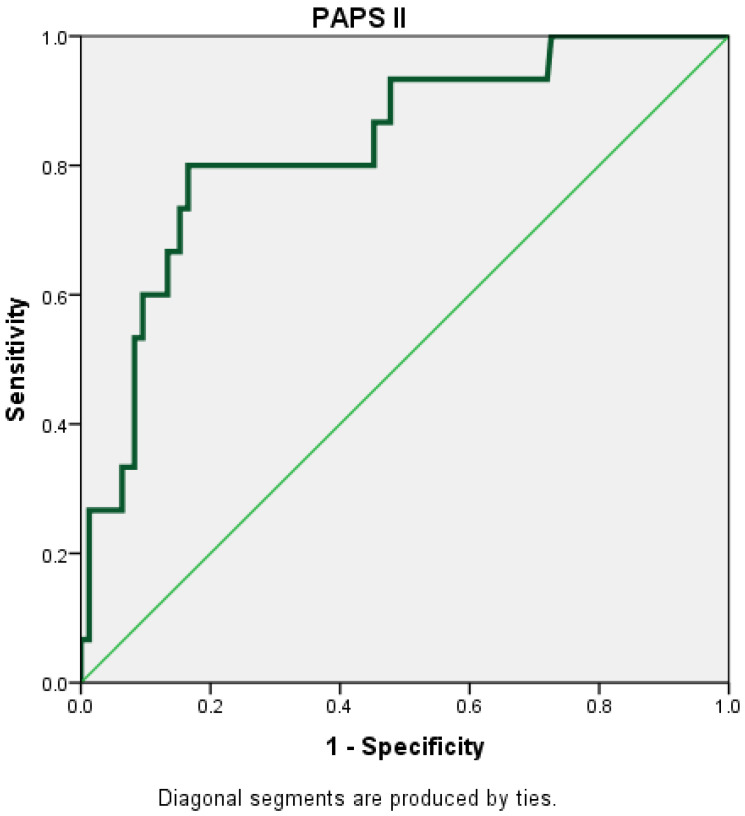
Receiver operating characteristic (ROC) curve for PAPS II.

The PAPS score II had the highest AUC (AUC = 0.830; 95% CI = 0.721–0.939) (Figure 3).

**Figure 4:**
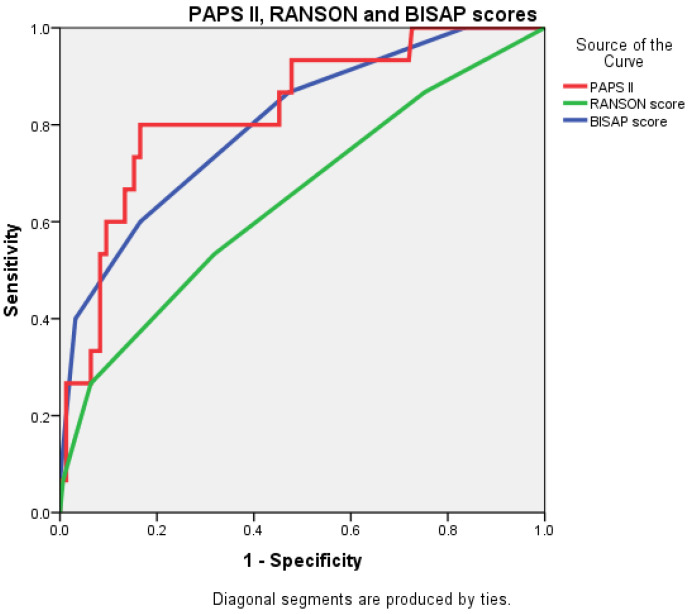
Receiver operating characteristic (ROC) curve comparing the predictive accuracy of BISAP, RANSON, and PAPS II.

**Figure 5:**
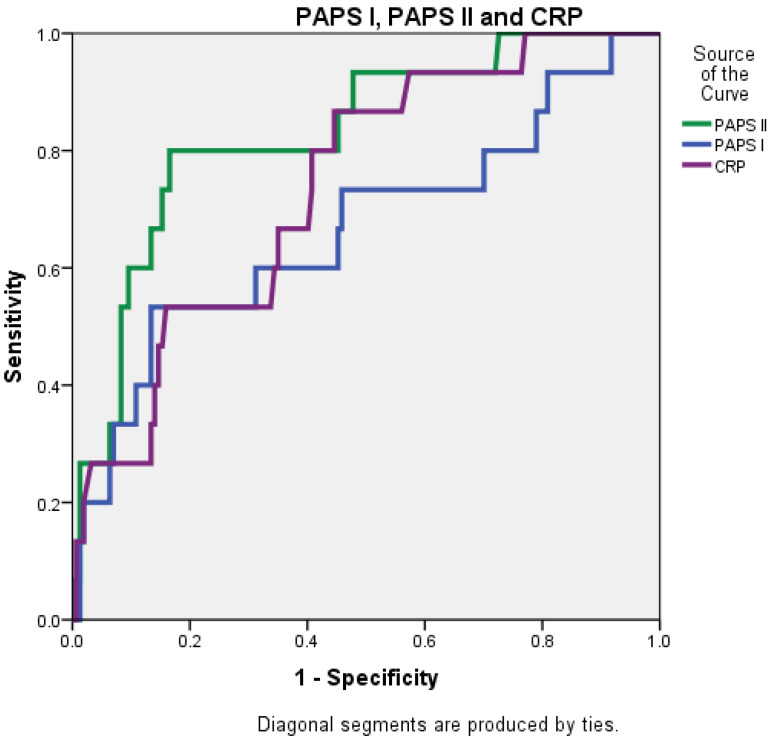
Receiver operating characteristic (ROC) curve comparing the predictive accuracy of PAPS I, PAPS II, and CRP.

The AUC value for PAPS II was 0.830 (0.721–0.939), significantly higher than that of the RANSON score at 0.647 (0.490–0.804) and closely followed by the BISAP score, which was 0.803 (0.684–0.922) (Figure 4 and Figure 5).

Considering the levels of the inflammatory markers analyzed and, implicitly, their predictive value, they could be influenced by the time elapsed from symptom onset to hospital admission; we analyzed the differences between patients admitted within the first 24 h, patients admitted between 24 and 72 h, and, respectively, admitted after 72 h from onset in both moderate and severe AP groups. Due to the fact that the CRP levels for patients admitted within 24 h showed non-significant differences compared with patients admitted between 24 and 72 h and over 72 h, neither in the group of those with moderate PA (*p* = 0.718) nor in the group of those with severe PA (*p* = 0.139), all the patients were considered for the study cohort.

Additionally, the ability of CRP to correctly identify the severity of AP was evaluated based on the interval between the start of symptoms and hospitalization. Thus, the threshold value of CRP for patients admitted within 24 h from symptom onset was 204 mg/L (AUC = 0.706; CI 95% = 0.575–0.837; sensitivity = 66.7% (29.9–92.5); specificity = 87.4% (78.5–93.5); PPV = 35.3% (21.0–52.9); NPV = 96.2% (90.9–98.5); accuracy = 85.4% (76.7–91.8)), and for patients who were admitted to hospital after 24 h from symptom onset, it was 235.0 mg/L (AUC = 0.795; CI 95% = 0.566–0.999; sensitivity = 66.7% (22.3–95.7); specificity = 88.6% (78.7–94.9); PPV = 33.3% (17.4–54.3); NPV = 96.9% (90.1–98.9); accuracy = 86.9% (77.1–93.5)).

## 4. Discussion

Finding a simple solution for triaging patients with acute pancreatitis into mild, moderate, or severe forms of the disease has always been a goal for medical staff in order to offer the best care for the patients and obtain the best outcome possible.

As a permanent quest for better scoring systems, this research theme is frequently encountered in the international literature. A retrospective study carried out at the Changhai Hospital elaborated on the new Chinese simple scoring system (CSSS). This score uses six factors: blood glucose, C-reactive protein, serum creatinine, lactate dehydrogenase, and the extent of pancreatic necrosis. The AUROC of the CSSS for predicting mortality was 0.838. The score proved to be the most accurate. The accuracy was lower for APACHE II, RANSON, MCTSI, and BISAP in this order [19]. Other Chinese studies improved a widely used score like Ranson and developed the modified Ranson score. This score was more accurate than the Ranson score in predicting severity, organ failure, and pancreatic infection or necrosis. This study stands for continuous research for improving severity scores [20].

The aim of our paper was to find predictive factors for acute pancreatitis in our hospital setting for the patients that are admitted to our facility and elaborate a new score of severity for AP.

Our study identified independent predictors for the severity of acute pancreatitis and developed three predictive models.

Patient characteristics may influence prognosis. In the case of AP, gender is not generally considered a predictor of AP severity in most studies. However, the geriatric population appears to develop more severe forms of AP, likely due to associated comorbidities and prolonged hospital stays [21,22]. Although our study did not identify a statistically significant difference between patients with mild to moderate AP and those with severe AP, advanced age has been consistently associated with poorer prognosis in the literature. A notable study conducted on a cohort of hospitalized patients in California demonstrated that individuals aged over 75 years had a risk of death more than 15 times higher during the first 14 days of hospitalization and over 22 times higher during the first 91 days, compared to patients younger than 35 years [23].

Consistent with worldwide patterns, our study found that alcohol consumption and biliary lithiasis were the main causes of acute pancreatitis. However, these etiologies were predominantly associated with mild to moderate forms of the disease. In contrast, a nonA-nonB etiology is linked to severe forms of AP, suggesting complex pathophysiological mechanisms or an amplified inflammatory response that warrants further investigation. An analysis of worldwide epidemiological trends in pancreatitis from 1990 to 2019 by Jiang et al. emphasizes the importance of alcohol and biliary lithiasis as the main cause of AP. According to their research, alcohol is the primary risk factor for AP and is responsible for a considerable number of fatalities, particularly in men aged 30 to 69. Additionally, the prevalence of gallstone-induced AP has been reported to increase with advancing age [2]. These results are consistent with our analysis of the prevalence of these etiologies; however, our findings about the correlation between severe AP and a nonA-nonB etiology may point to possible regional variations or cohort-specific features.

In the studied patient cohort, we identified a weak but statistically significant positive correlation between the severity of acute pancreatitis (AP) episodes and the presence of diabetes mellitus, with a Spearman’s rho correlation coefficient of 0.183 and *p* = 0.016. These results align with previous studies that have suggested diabetes mellitus as a risk factor for severe AP progression, likely due to mechanisms involving metabolic imbalances and exacerbated inflammation. Other studies have shown that patients with both diabetes and acute pancreatitis tend to experience more complications, prolonged hospital stays, an increased risk of renal failure, and higher mortality rates [24]. For instance, a study conducted by Huh et al. on 201 AP patients, including 54 with type II diabetes, demonstrated that diabetic patients encountered higher values for severity scores, such as Ranson and BISAP. They also developed moderate or severe forms of AP as far as the Atlanta classification is concerned, compared to non-diabetic individuals who were mostly registered with mild and less moderate forms of AP and built up lower values for severity scores. Additionally, diabetic patients exhibited a higher prevalence of intensive care unit admissions (31.5% vs. 18.4%) and significantly increased mortality rates (16.7% vs. 3.4%, *p* = 0.001) [25]. Also, Kikuta et al. analyzed a group of 1954 AP patients in a national study conducted in Japan and compared diabetic and non-diabetic groups, paying special attention to the first 72 h. The resulting observation highlights the fact that patients with diabetes had higher rates of cardiovascular failure (*p* < 0.05) and renal failure (*p* < 0.01), especially in the first 48 h, underscoring the early impact of diabetes on AP prognosis [26]. However, the relatively modest correlation obtained in our study highlights that diabetes is most likely only one of the multiple factors involved in determining the severity of the disease, which makes further research necessary to clarify both the mechanisms underlying this relationship and the clinical relevance.

A prior history of AP is valuable information, particularly for primary diagnosis, although, in our study, previous episodes did not directly influence disease severity.

Correlation analysis between the severity of acute pancreatitis and clinical-biological parameters revealed significant associations, emphasizing the interplay between inflammatory processes, etiological factors, and clinical manifestations. These findings, detailed in Table 5, highlight that certain biological and etiological variables are significantly correlated with disease severity, offering insights into the underlying pathophysiological mechanisms and potential pathways for clinical risk stratification.

In the study conducted in our hospital, we did not prove the existence of a statistically significant relationship correlating the increased value of the hematocrit with the severity level of the acute pancreatitis episode. However, researchers from the University of Calgary in Canada established, after studying a cohort of 200 patients, that hematocrit from admission is an early predictor of severe pancreatitis. Specifically, they highlighted that a hematocrit value above 50% is associated with a high risk of developing a severe form of acute pancreatitis and also that an increase of only 5% of the hematocrit is a good predictor of disease severity, period of hospitalization, likelihood of admission to intensive care and necrosis [27]

The presence of high pain levels on the VAS scale in cases of severe AP suggests that clinical symptom intensity may serve as an indicator of severe disease. We chose to study this factor because acute abdominal pain is one of the most common symptoms, leading patients with AP to present to the emergency room. Pain is costly for the medical system. In the United States, the cost is estimated to be between USD 560 and 635 billion per year, higher than the cost of heart disease and cancer treatment [28,29]. Patients with severe AP, who were included in our study, reported a VAS score of 10.0 (IQR: 9.0–10.0), which is significantly higher than the value obtained in patients diagnosed with mild and moderate forms of the disease (8.0, IQR: 7.0–10.0, *p* = 0.003). Subsequently, this result was also supported by the obtaining of a significant positive correlation between VAS scores and disease severity (r = 0.229, *p* = 0.003), which highlights the usefulness of its use in assessing the symptomatology of patients upon hospitalization. To analyze the management of pain in AP using the VAS scale to assess intensity, Cai et al. performed a meta-analysis that demonstrated that the use of specific analgesia significantly reduces the need for emergency analgesics (OR 0.36, 95%, CI 0.21–0.60, *p* = 0.0001). At the same time, the use of specific analgesia also led to an improvement in VAS scores in the first 24 h (WMD 18.46, 95%, CI 0.84–36.07, *p* = 0.04) [28]. The frequency of VAS measurements and the use of specific analgesia, as methodological differences between the presented meta-analysis and our study, could explain the variations in the results and, at the same time, could constitute the foundation that advocates the need for standardized protocols for assessing pain and integrating analgesic management into the stratification of PA severity.

CRP is described in the literature as a fine predictor for the severity of AP. Many studies take into account evaluating CRP levels at different intervals, and there is a variety of cut-offs mentioned: 120 mg/L, 150 mg/L, 190 mg/L, and interval change in CRP level of 90 mg/L [30,31]. The CRP value, measured exclusively at admission to the emergency department, demonstrates a moderate positive correlation with the severity of AP (r = 0.233, *p* = 0.002). The result also highlights the usefulness of CRP as an inflammatory marker with an important initial role in risk stratification at the time of patient admission to the emergency department. Comparatively, in the specialized medical literature, Ahmad et al. have highlighted, by evaluating CRP both at admission and at 48 h, that the predictive value of CRP at the time of admission is reduced (AUC = 0.54, *p* = 0.74). Although its performance increased at the reassessment performed at 48 h (AUC = 0.70, *p* = 0.007), the positive predictive value for the cut-offs of 150 mg/L and 190 mg/L, remains modest (30 and 28%, respectively), thus indicating a limited utility in predicting complicated forms of AP of this marker. However, CRP could be used to exclude severe cases due to the existence of a considerably high negative predictive value (>80%) [30]. Although the resulting differences could be due to different methodologies, both studies highlight the limitations of CRP as a single marker of AP severity and suggest that the use of multifactorial scores or other additional biomarkers may be beneficial. This conclusion is supported by other valuable sources in the literature that strongly argue against the use of a single severity predictor in AP risk stratification [32,33]. In the studied cohort, no statistically significant differences were found according to the symptom onset for CRP levels for patients admitted within 24 h compared with patients who were admitted to hospital after 24 h from symptom onset. Thus, we believe that the moment of hospital admission represents the peak of symptom severity in the disease evolution up to that point (that led the patients to seek medical help). We also note that well-established scoring systems such as Ranson, BISAP, and APACHE do not take symptom onset into account, and the severity of the disease is assessed based on the patient’s status at the time of admission [34,35,36].

Fluids administered during the first 24 h and biological parameters such as pancreatic enzymes and ALT did not demonstrate a significant impact on severity prediction in this study. Over time, there have been various prospective and retrospective studies that have attempted to clearly and practically demonstrate the benefits of fluid resuscitation. A study published by Gardner et al. investigated the impact of early intravenous resuscitation rates within the first 24 h of hospital presentation on the clinical outcome of patients with AP. Therefore, patients with severe acute pancreatitis who initially received aggressive fluid resuscitation had lower morbidity and mortality than those who received less aggressive fluid resuscitation or no fluid resuscitation [37]. Although intravenous fluid administration is a central pillar of AP treatment, numerous studies have shown that there is no clear consensus on the optimal volume, type of fluid, or rate of administration [38]. For example, in a study of approximately 249 patients diagnosed with AP, the clinical outcome of patients who received an aggressive fluid resuscitation approach was compared with the clinical outcome of those who received a moderate fluid resuscitation approach. Thus, no differences in the incidence of moderate-severe or severe forms were observed in terms of length of hospital stay. Unfortunately, the study was stopped prematurely due to high rates of fluid overload among patients who received aggressive volume resuscitation (20% compared with 6%) [39]. Therefore, the absence of a standardized protocol on how to perform volume resuscitation may represent a starting point for future scientific research conducted with the aim of improving the clinical outcome of patients with PA, especially those with severe forms.

Using the predictive factors studied in our cohort, we elaborated three predictive models for the severity of acute pancreatitis. Among the three models studied (CRP, PAPS score I, and PAPS score II), the PAPS score II demonstrated the highest AUC

For our cohort, the Ranson score proved a sensibility of 53.3%, a specificity of 68.2%, a PPV of 13.8%, an NPV of 93.9%, and an accuracy of 0.647. In another study, elaborated by Scott Tenner et al., the sensitivity of the Ranson score ranges from 40% to 88%, the specificity ranges between 43% and 90%, the PPV is 50%, and the NPV approximates to 90%. Sensitivity, specificity, and NPV are comparable in both studies. The high value of the NPV leads to the use of the Ranson score to exclude patients with severe forms of acute pancreatitis [40]. Another review paper published in Singapore in 2021 argues that the Ranson score has a comparable accuracy to new scoring systems [41].

The BISAP score calculated for the patients with acute pancreatitis evaluated in our study encountered a 60% sensitivity, 83.4% specificity, 25.7% PPV, and 95.6 NPV for predicting severity and has an accuracy of 80.8%. A study conducted by Anum Arif et al. in 2019 showed values for sensitivity (69.2%) and specificity (77.8%) for BISAP scores comparable with the ones in our study [42].

There are some studies that encounter better specificity and sensitivity for the severity of disease of the Ranson score and BISAP score. A study published in 2024 by Jianpeng Zu et al. presents, following a meta-analysis, a pooled sensitivity for severity for the Ranson score and BISAP score of 95% and 67%, a pooled specificity for both scores of 74% and 95%, and a pooled accuracy of Ranson score and BISAP score of 95% and 94% [43].

Comparing the PAPS score II with the Ranson score and the BISAP score, we emphasize the following. While the NPV has comparable values for all three scores, the Ranson score has the lowest PPV and BISAP score, and the PAPS score II has close values for PPV. The highest specificity is proved by the BISAP score. Nevertheless, the highest sensitivity belongs to PAPS score II.

The PAPS score II, in comparison with the two other recognized scoring systems, the Ranson score and BISAP score, had the best accuracy in our study.

PAPS score II is easy to use, memorize if necessary, and cost and time-efficient, even more than the BISAP score. The PAPS score II uses four parameters that are immediately available in the ER setting, while BISAP uses eight parameters that are also immediately available in the ER. The Ranson score uses 11 parameters with variable availability in the hospital setting and needs 48 h to be determined.

The PAPS II score, like any other severity score, can be used independently, as it has been shown to have good specificity in identifying cases of severe acute pancreatitis. However, using it alongside other established scores can provide extremely valuable additional information, providing the medical team with a much better perspective of the patient. The combined use of multiple scores can bring improvements in terms of both the accuracy of clinical decisions and optimize therapeutic management.

The PAPS score II is considered highly applicable in clinical practice.

The primary limitation of this study is the relatively small sample size and the limited number of severe AP cases included. Additionally, the retrospective study design inherently limits control over the quality and consistency of data collection. Furthermore, the predictive score was not compared with another widely used scoring system, such as the APACHE II score. Although the inclusion of APACHE II would have been ideal, the limited availability of data for patients outside the ICU necessitated the use of alternative, faster, and easier-to-apply scores. Our goal was to identify a predictive model that could be applied at the time of patient presentation without being constrained by parameters that are not always available or affordable.

Elevated levels of C-reactive protein (CRP) and white blood cell counts (WBCs), while significant, are not specific to AP and may occur in various conditions, including other infections. Moreover, higher CRP levels could be associated with underlying liver disease, which is common among patients with alcoholic etiology of AP.

## 5. Conclusions

Our study identified these parameters as being associated with the severity of AP: etiology nonA-nonB, presence of diabetes mellitus, pain VAS, WBC, and CRP levels. A score using these parameters was elaborated on to identify patients with the potential for severe AP. For these patients, a CT scan (unless already done) should be performed, and admission to the ICU should be arranged. This score is easy to use, affordable for ER or any other ward or medical institution, and easily applicable. We are interested in comparing this score with other widely used scores like Apache scores in the future. Also, we intend to validate this score on an extensive number of patients and in other medical facilities, as well as other regions.

## Figures and Tables

**Table 1:** Patient characteristics.

Parameters	Mild and Moderate AP (157 Patients)	Severe AP (15 Patients)	*p*-Value
Gender N (%)			0.588
male	95 (60.5)	8 (53.3)	
female	62 (39.5)	7 (46.7)	
Age (years) M ± SD	55.9 ± 16.2	59.7 ± 18.0	0.388
BMI (kg/m^2^) Q2 (Q1–Q3)	25.0 (22.5–29.2)	26.0 (23.0–31.0)	0.497
Diabetes N (%)	31 (19.7)	7 (46.7)	0.016
Previous episodes AP N (%)	45 (28.7)	4 (26.7)	0.870
INR Q2 (Q1–Q3) (Reference Range: 0.800–1.140)	1.1 (1.0–1.2)	1.1 (1.0–1.2)	0.961
ALT 1 Q2 (Q1–Q3) (Reference Range: 0–50 U/L)	120.5 (59.2–309.2)		0.310
VAS 0 Q2 (Q1–Q3)	8.0 (7.0–10.0)	10.0 (9.0–10.0)	0.003
WBC 1 Q2 (Q1–Q3) (Reference Range: 4–10× m/mm^3^)	12,280 (9350–15,700)	19,080 (10,100–21,800)	0.037
Hematocrit 1 (%) (Reference Range: 33–54%)	43.3 ± 6.7	43.5 ± 7.5	0.879
Amylase 24 h Q2 (Q1–Q3) (Reference Range: 28–100 U/L)	683 (239.5–1257)	1023 (381–1782)	0.241
Lipase 24 h Q2 (Q1–Q3) (Reference Range: 8–78 U/L)	6275 (2280–8850)	6196 (3468–9862)	0.706
Calcium Q2 (Q1–Q3) (Reference Range: 8.2–10.7 mg/dL)	8.7 (8.1–9.1)	8.8 (6.8–9.0)	0.236
C Reactive Protein (mg/L) Q2 (Q1–Q3) (Reference Range: 0–5 mg/L)	85.0 (33.5–171.5)	223.0 (126.0–320.0)	0.002
Fluids administered 24 h (mL) Q2 (Q1–Q3)	3000 (2500–4000)	3000 (2000–4000)	0.842

M = mean; SD = standard deviation< Q2 = median; Q1 = 25 quartile; Q3 = 75 quartile; Etiology nonA-nonB = etiology non-alcoholic (unique)-non-biliary (any etiology except unique alcoholic or biliary etiology).

**Table 2:** Distribution of etiology in the studied cohort.

Etiology	Frequency	Percent (%)
Biliary lithiasis	72	40.7
Alcohol	61	34.5
Hypertriglyceridemia	7	4.0
Mixt-biliary + alcohol	9	5.1
Mixt-alcohol + hypertriglyceridemia	9	5.1
Other	10	5.6
Idiopathic	4	2.3

**Table 3:** Type of etiology.

Etiology	Frequency	Percent
Unique etiology	154	87.0
Multiple etiology	18	10.2

**Table 4:** Biliary etiology.

Etiology	Frequency	Percent
Biliary lithiasis	71	40.1
Mixt biliary	9	5.1
Non-biliary	92	52.0

**Table 5:** Correlation between AP severity and biological parameters.

Etiology	Spearman’s Rho Correlation Coefficient	*p*-Value
**Gender**		
Male	−0.041	0.591
Female	0.041	0.591
Age	0.045	0.559
**Etiology**		
Biliary	−0.095	0.214
Alcohol	−0.100	0.192
nonA-nonB	0.226	0.003
BMI	0.052	0.499
Diabetes	0.183	0.016
Previous episodes AP	−0.012	0.871
INR	0.004	0.961
ALT	−0.078	0.312
VAS 0	0.229	0.003
WBC	0.159	0.037
Hematocrit	−0.019	0.804
Amylase	0.090	0.242
Lipase	0.029	0.707
Calcium	−0.188	0.014
C Reactive Protein	0.233	0.002
Fluids 24 h	−0.015	0.842

**Table 6:** Logistic regression analysis of independent predictors of AP severity.

Parameter	B	SE	Wald	*p*-Value	OR	95% CI Lower	95% CI Upper
**Gender**							
Male	−0.293	0.543	0.292	0.589	0.746	0.257	2.161
Female	0.293	0.543	0.292	0.589	1.341	0.463	3.884
Age	0.014	0.017	0.748	0.387	1.015	0.982	1.048
**Etiology**							
Biliary	−0.742	0.606	1.503	0.220	0.476	0.145	1.560
Alcohol	−0.852	0.666	1.633	0.201	0.627	0.116	0.575
nonA-nonB	1.536	0.555	7.657	0.006	0.006	1.565	13.786
BMI	0.021	0.050	0.182	0.670	1.021	0.926	1.126
Diabetes	1.269	0.555	5.226	0.022	3.556	1.198	10.555
Previous episodes AP	−0.100	0.610	0.027	0.870	0.905	0.274	2.991
INR	−0.773	1.235	.391	0.532	0.462	0.041	5.199
ALT	−0.002	0.002	1.625	0.202	0.998	0.994	1.001
VAS 0	0.742	0.292	6.450	0.011	2.099	1.184	3.721
WBC	0.000	0.000	7.050	0.008	1.000	1.000	1.000
Hematocrit	0.006	0.040	0.023	0.878	1.006	0.930	1.089
Amylase	0.000	0.000	0.992	0.319	1.000	1.000	1.001
Lipase	0.000	0.000	0.007	0.933	1.000	1.000	1.000
Calcium	−0.642	0.272	5.586	0.018	0.526	0.309	0.896
C Reactive Protein	0.009	0.003	10.654	0.001	1.009	1.003	1.014
Fluids 24 h	0.000	0.000	0.002	0.968	1.000	1.000	1.000

B = beta coefficient; SE = standard error; CI = confidence interval.

**Table 7:** Logistic regression models predicting AP severity.

Parameter	B	SE	β	*p*-Value	95% CI Lower	95% CI Upper
**Model 1**						
C-Reactive Protein	0.001	0.000	0.270	<0.001	0.000	0.001
**Model 2**						
nonA-nonB etiology	0.142	0.049	0.210	0.004	0.046	0.238
WBC	0.001	0.000	0.159	0.032	0.000	0.000
VAS 0	0.025	0.011	0.165	0.026	0.003	0.046
CA	−0.057	0.024	−0.174	0.016	−0.104	−0.011
**Model 3**						
C-reactive protein	0.001	0.000	0.185	0.014	0.000	0.001
nonA-nonB etiology	0.135	0.049	0.201	0.006	0.039	0.231
VAS 0	0.025	0.011	0.166	0.024	0.003	0.046
CA	−0.022	0.001	−0.146	0.048	0.000	−0.043

B = unstandardized coefficient; SE = standard error; β = standardized coefficient; CI = confidence interval for B.

**Table 8:** The performance of CRP, PAPS score I, PAPS score II, BISAP score, and RANSON score to predict the severity of AP.

Models	AUC (95% CI)	Cut-Off Value	Se% (95% CI)	Sp% (95% CI)	PPV% (95% CI)	NPV (95% CI)	Accuracy% (95% CI)
CRP	0.739 (0.619–0.859)	221.5	53.3 (26.6–78.7)	84.1 (77.4–89.4)	24.2 (15.0–36.7)	95.0 (91.6–97.0)	81.4 (74.8–86.9)
PAPS score I	0.667 (0.502–0.832)	20.5	53.3 (26.6–78.7)	86.6 (80.3–91.5)	27.6 (17.0–41.4)	95.1 (91.9–97.1)	83.7 (77.3–88.9)
PAPS score II	0.830 (0.721–0.939)	1.248	80.0 (51.9–95.7)	80.9 (73.9–86.7)	28.6 (21.0–37.6)	97.7 (93.9–99.2)	80.8 (74.1–86.4)
BISAP score	0.803 (0.684–0.922)	2.5	60.0 (32.3–83.7)	83.4 (76.7–88.9)	25.7 (16.8–37.3)	95.6 (92.1–97.6)	81.4 (74.8–86.9)
RANSON score	0.647 (0.490–0.804)	1.5	53.3 (26.6–78.7)	68.2 (60.3–75.4)	13.8 (8.6–21.3)	93.9 (89.9–96.4)	66.9 (59.3–73.8)

## Data Availability

This article does not include any additional primary data besides the information already presented in the case report section.
